# The initial impact of COVID-19 and policy responses on household incomes

**DOI:** 10.1093/oxrep/graa024

**Published:** 2020-06-26

**Authors:** Mike Brewer, Laura Gardiner

**Keywords:** COVID-19, poverty, tax and benefit system, welfare

## Abstract

As soon as the scale of the coronavirus shock to the economy became clear, the UK government introduced three policies to protect directly household incomes: a Job Retention Scheme, to pay the wages of employees who were temporarily furloughed; a Self-Employment Income Support Scheme, to give grants to established self-employed people whose businesses had been affected; and a package of increases to entitlements to social security benefits, with Universal Credit at the core, that bolstered the UK’s means-tested ‘safety net’. This paper analyses the design and beneficiaries of these policies and, given the distributional pattern of the labour market shock, considers the emerging overall impact on living standards, particularly of low-income households.

**JEL code**: I38, J38.

[1] Mike Brewer is Chief Economist at the Resolution Foundation and Professor of Economics at the University of Essex, and Laura Gardiner is Research Director at the Resolution Foundation. Brewer acknowledges support from the UK Economic and Social Research Council (ESRC) through the Research Centre on Micro-Social Change (MiSoC) at the University of Essex (award number ES/S012486/1).
© The Author(s) 2020. Published by Oxford University Press.
This is an Open Access article distributed under the terms of the Creative Commons Attribution License (http://creativecommons.org/licenses/by/4.0/), which permits unrestricted reuse, distribution, and reproduction in any medium, provided the original work is properly cited.

## 1. Introduction

The UK is in the midst of the biggest shock to economic activity that it has experienced for hundreds of years, one that is rooted in the labour market, driven by the shutting down of certain sectors of the economy and the effects of social distancing rules. At the time of writing, the Bank of England is expecting the economy to shrink by 25 per cent in the second quarter of 2020 (and by 14 per cent for the year as a whole). The Office for Budget Responsibility (OBR) is expecting a 35 per cent GDP fall in the second quarter.

This paper assesses how the crisis is affecting, or is likely to affect, household incomes, and particularly low-income households. In section 2, we assess the three core components of the UK government's strategy to directly protect household incomes: the social security system (including the changes announced once the crisis began), and the new Coronavirus Job Retention Scheme and the Self-Employment Income Support Scheme (we limit our attention to policies announced by the UK government and do not discuss the (very minor) additional policies announced by the devolved administrations). Using microsimulation methods, we show how well these changes protect households from the financial implications of unemployment. In section 3, we discuss the emerging evidence on the impact of the crisis on the distribution of living standards. Although there is not yet a consensus, it seems that the fall in household incomes is more evenly spread across the income distribution than the loss of jobs is distributed across the earnings distribution, and may even be greater among those on higher (pre-crisis) incomes. But we also show that other indicators of financial well-being or living standards than income paint a more worrying story about the ways that the crisis is affecting low-income households.[2] We conclude in section 4.

One limitation of this article is that, given the evidence and data sources that we draw on, it may not reflect the experiences of those people facing the worst disadvantage. We do not make any attempt to analyse, for example, how the analysis is affecting the homeless population. Attention has also been drawn to those whose migration status means that they are not able to claim means-tested social security benefits because they are in the UK on the condition that

[2] The risk of an article such as this is that it could soon be out of date; Brewer (2020) will contain a more up-to-date summary of the UK evidence.

they have no recourse to public funds (Gower and Kennedy, 2020). Much of the near real-time research that is being done during the coronavirus crisis is through online surveys, and so will miss the digitally excluded. We acknowledge these drawbacks, as well as recognizing that behind the data we describe lie millions of households experiencing genuine hardship and financial strain.

## 2. The initial UK government response to the coronavirus

### 2.1. Details of the measures to protect household incomes directly

Like other countries, the UK government has responded to the current crisis with a set of policies unprecedented in their scope and cost. Indeed, the UK's has been one of the largest fiscal responses among advanced economies (Hughes *et al*., 2020). Here, however, we focus on the three elements relating more directly to individual and household incomes: the Coronavirus Job Retention Scheme (JRS), the Self-Employment Income Support Scheme (SEISS), and changes to the social security system.

The JRS takes the unprecedented step of directly supporting employers to pay the wage bills of employees 'furloughed' without work to do. From April to July, the JRS paid 80 per cent of pre-coronavirus wages, up to £2,500 a month, and on current plans it will continue to pay 80 per cent in August to October, but with a lower cap and some cost-sharing with employers (HMRC, 2020). The idea is that when economic activity picks up, furloughed employees will go back to their pre-crisis work, but this is not a formal condition placed on employers. By mid-June, around 9 million jobs had been supported at some point by the scheme (HMRC, n.d.). The JRS is the largest single part of the government's response, with the total (gross) cost currently estimated to be around £60 billion (OBR, 2020).

The SEISS can be seen as a parallel scheme for the self-employed, in that it provides similar levels of income replacement over two sets of grants intended to cover a 6-month period. It is restricted to unincorporated self-employed businesses; those with profits above £50,000 are excluded, as are the newly self-employed (i.e. those without at least one complete tax return in the 3 years up to 2018/19, although there is a separate scheme in Scotland to help this group). By mid-June, 2.6 million claims for the initial grant payment had been made (HMRC, n.d.). The expected total gross cost is £15 billion (OBR, 2020).

The government has also announced major changes to the social security system. [3] The most important was a £20 per week increase in the standard allowance of Universal Credit (UC), an increase that was mirrored in the Working Tax Credit, but not in the contributory ('new-style' Jobseeker's Allowance (JSA) and Employment and Support Allowance (ESA)) or other 'legacy' benefits. This change aligns the main adult rate of benefits for an unemployed single adult to the level of Statutory Sick Pay, and means it is at its highest ever level in real terms, and at its highest level relative to average earnings since 1998/99 (see Figure 1). It is noteworthy that the size of the COVID-19 rise in UC was the same for all family types, with no additional help offered to families with children.[4] In addition, Local Housing Allowance (LHA) rates (which determine caps on housing support in UC and Housing Benefit for private-sector tenants) were increased, particularly favouring areas in which rents have grown rapidly since 2012 (Judge and Pacitti, 2020). We discuss more who gains from this reform in the next section, but we note now that the detailed rules about UC entitlement mean that the UC increase will not be felt in full by recipients who are affected by the benefit cap (although some of the newly unemployed will benefit from a 9-month grace period), and the LHA increases do not benefit renters in those parts of London that are affected by a national cap on LHA rates. These measures followed some technical changes in the March 2020 Budget that help the self-employed, including the suspension of the minimum income floor in UC (which in effect increases the amount that very low-income self-employed workers can claim), and the removal of the 7-day waiting period that restricted access to contributory ESA (which self-employed people can claim when sick or self-isolating). Local authorities were also given extra funds to provide help with Council Tax bills and to enhance local welfare provision. At the time of announcing the measures, the government said that the boost to social security benefits and tax

[3] We do not attempt to give a thorough guide to the social security system in the UK. It will help to know that the two most important benefits for those who are made unemployed are 'contributory' or 'new style' Jobseeker's Allowance (JSA), and Universal Credit. 'New style' JSA is available to former employees who have made sufficient National Insurance contributions, and is payable without a means test (i.e. regardless of how much an individual has in savings or whether they are living with a partner with their own earnings). It is paid at a low flat rate that does not try to reflect any additional needs that a person might have, including the presence of a partner or children. Those formerly self-employed are not able to claim. Universal Credit, like all means-tested benefits in the UK, does not require someone to have made sufficient National Insurance contributions, and entitlement is usually higher for families with children or health or disability needs, for example, than those without. But awards are means tested against the total income of the family and any savings. Different benefits are available to those who cannot work through ill-health or disability, and we do not discuss those further.

[4] A scheme was introduced to give vouchers or food parcels to families whose children would have received free school meals had they been attending school, but this is simply maintaining the pre-pandemic levels of support, rather than providing additional help to those with children.

credits would apply only during the 2020/21 financial year, and the latest estimate is that it will cost £8 billion (OBR, 2020).

**Figure 1:** The value of the main rate of unemployment-related benefit over time for a single adult

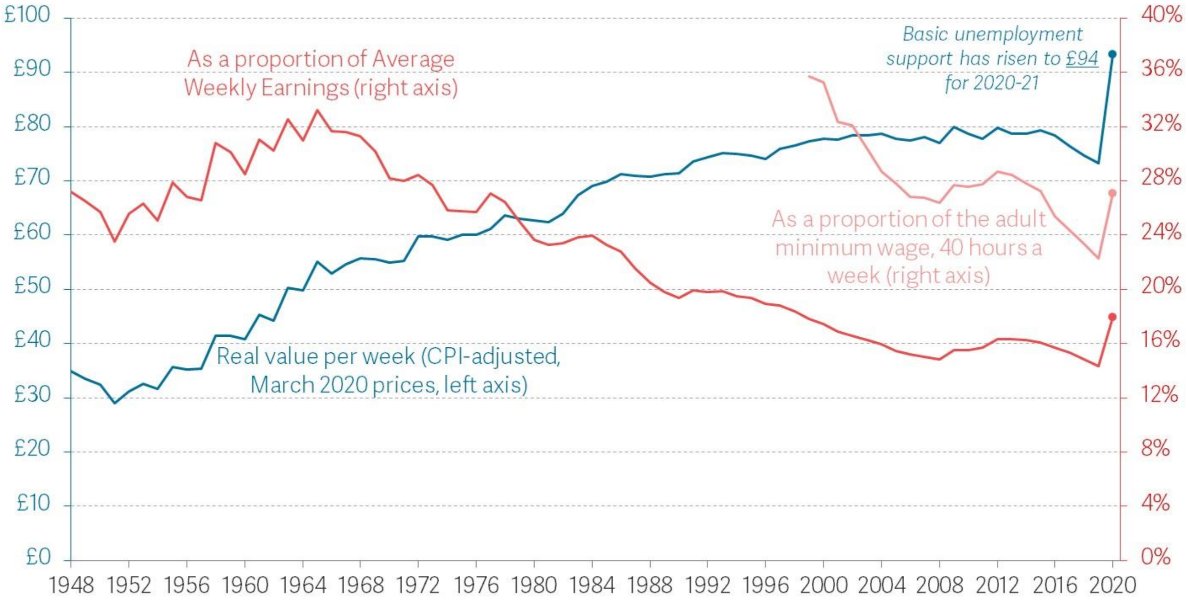

Notes: Full-time earnings on the minimum wage calculated based on a 40-hour week.
Source: Figure 16 of Brewer and Handscomb (2020).

## 2.2. Assessment of what the measures have done to the amount of income protection provided by the state

Given that the impact of COVID-19 on household incomes is operating primarily through the labour market, it is important to understand how the tax and benefit system, together with the new policies announced in response to the pandemic, act to protect household incomes in the event of job loss.

Figure 2, from Brewer and Handscomb (2020), shows how replacement rates (these report how much income someone would have if they lost their job expressed as a proportion of the income they had when in work) vary by personal and family characteristics in the 2020–21 system after the new measures, as well as showing median replacement rates under the tax and benefit system that we were due to have from April 2020 in the absence of the emergency benefit increases (Adam *et al*. (2020) have similar analysis). It shows a great deal of variation in the extent to which the social security system protects people from the financial consequences of unemployment. This reflects at least three factors. First, people's entitlement to benefits if they do not work will depend on their family circumstances, meaning that two people on similar levels of earning may face different replacement rates (this can be seen in the low replacement rates for single adults without children who have low entitlements to benefits compared to those with children, especially for those under 25). Second, benefits paid in the event of unemployment in the UK are paid at a (low) flat rate, irrespective of previous earnings, and so those on higher earnings tend to face lower replacement rates. Third, asset tests in the calculation of UC mean that families with more than £16,000 of savings will miss out on this support entirely (as shown in, for example, Figure 19 in Brewer and Handscomb (2020)). Such tests have their justifications, but pose challenges to the social security system's ability to function as a safety net during such a large and rapid economic shock. [5]

Figure 2 shows that the impact of the coronavirus reforms was to increase the median replacement rate across all workers from 50 to 53 per cent. However, the fact that the increase to UC was a flat-rate £20 per week for all family types means that the maximum UC entitlement for a couple aged 25 or over with two children rose by just 11 per cent (and median replacement rates for workers in these families improved by just 3 ppts), whereas the rate for a single adult under 25 rose by 36 per cent on its 2019 value (and the median replacement rate for this group improved by 8 ppts).

[5] International comparisons of replacement rates are difficult and can depend on family circumstances and the duration of unemployment, but Organisation for Economic Co-operation and Development (OECD) comparisons do show that the UK ranks towards the bottom of international league tables in terms of the amount of income protection provided by benefits in the event of unemployment: see https://data.oecd.org/benwage/benefits-in-unemployment-share-of-previous-income.htm#indicator-chart, accessed 22 May 2020, for example, which looks at replacement rates for single adults without children paid at 67 per cent of the national average.

**Figure 2:** Family income replacement rates when earner stops working and claims benefits by selected characteristics, latest policy: UK, 2020–21

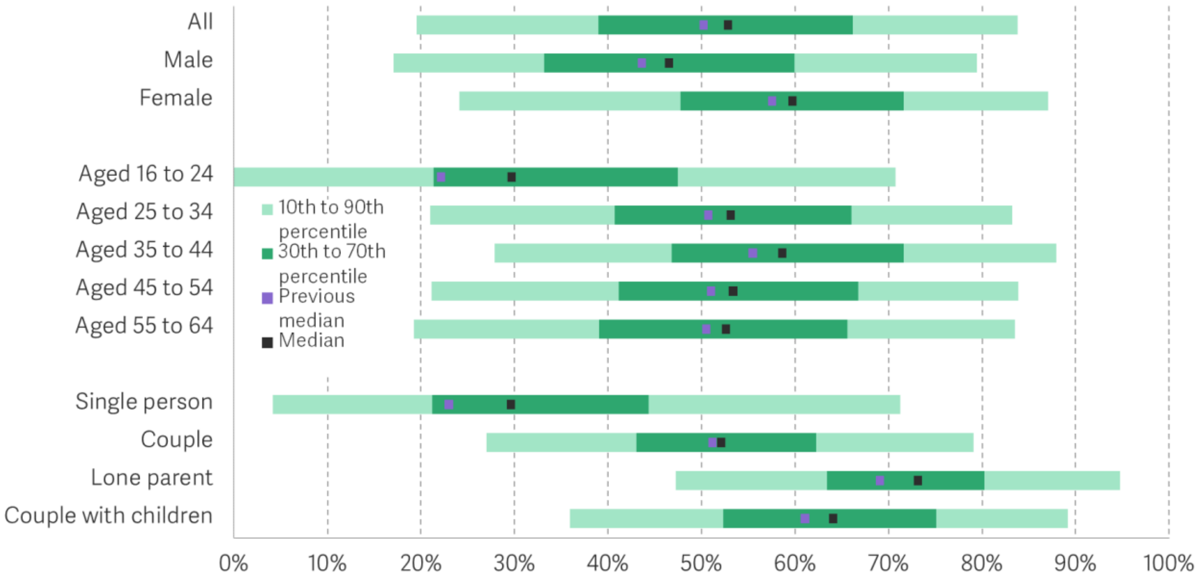

*Notes*: Replacement rates shown for benefit unit income before housing costs, for adults aged 16–64 who stop working and then claim benefits as entitled. Partner income held constant. Full roll-out of UC and full take-up of benefits assumed.

*Source*: Brewer and Handscomb (2020).

Figure 20 of Brewer and Handscomb (2020) shows that the degree of income protection provided by the social security system is significantly weaker than that provided by the JRS and SEISS. In a world where all employees are placed on the JRS and all self-employed workers receive the SEISS, then the median replacement rate is 91 per cent (it is greater than 80 per cent partly because many people will pay lower effective tax rates after a 20 per cent fall in earnings, partly because some people on the JRS can also claim means-tested support through UC, and partly because of the earnings of working partners for those in couples), compared to 53 per cent without it). This highlights a very important issue of fairness: that those former employees who are not currently benefiting from the JRS—perhaps because they were made redundant, or were ineligible because they had only just started a new job as the crisis hit, or because their employer went bust—will be typically experiencing a far greater hit to their incomes than those (relatively) lucky enough to be furloughed. It also suggests that, as the JRS scheme is reformed during the second half of 2020, any currently furloughed workers who are made redundant may experience large falls in their family income.

## 3. What is (likely to be) the impact of COVID-19 on household incomes?

High-quality data on household incomes tends to be released with long lags and so official estimates of income poverty and inequality for current (at the time of writing) financial year, 2020/21, will not be available until March 2022.

Until then, we have to rely on other sources. We first summarize what is known about the distribution of the initial impact on jobs and earnings based on data from online surveys of individuals or households. We then consider what is known about the impact of the policy measures. After that, we summarize attempts made to combine these and ‘nowcast’ (see Navicke *et al.*, 2014) the entire distribution in a way that allows researchers to isolate the impact of the crisis on the distribution of income. Finally, we look at what we can learn from measures of living standards and financial wellbeing other than income.

Before doing that, it is worth looking back at how low-income households have fared in previous recessions and economic crises. In the UK recessions of the mid-1970s, the early 1980s, and the early 1990s, the number of individuals in relative poverty—defined as living in a household with less than 60 per cent of median income—fell, driven by large reductions in pensioner poverty (Muriel and Sibieta, 2009). This happened because pensioners get most of their income from sources other than the labour market, and so they tend to move up the income distribution when unemployment rises and earnings from the labour market fall, reducing the number below a floating poverty line. Indicators of poverty that use an anchored poverty threshold increased in all three of these recessions; this is what most people would predict: during recessions and crises, living standards, as a whole, fall.

In the recession that followed the global financial crisis, relative poverty fell very slightly from 2007/08 to 2012/13, with falls among pensioners that were almost entirely offset by rises among other groups. Unusually, poverty assessed with an anchored poverty line hardly changed from 2007/08 to 2010/11, but then started to rise in 2011/12, especially for those below the state pension age, due to the austerity measures that followed. This reminds us that the policy responses to crises determine the impact on the vulnerable as much as the initial economic shock.

### 3.1. The distributional impact of the initial labour market shock

At the time of writing, the usual official estimates of the employment and unemployment rate were still somewhat dated. Timelier data give a better sense of the scale of the impact of restrictions on economic activity from late March on the jobs market, though, with a fall of 612,000 in employee jobs captured by PAYE systems between March and May, an 1.6 million increase (up to 2.8 million) in the claimant count measure of unemployment-related benefit recipients, and a more-than-halving of vacancy numbers (by early May). These numbers, although representing extremely large changes in just 2 months, are without doubt much lower than they would have been had the JRS not been in existence, which has supported 9 million jobs at some point in that same time period. More granular week-by-week data from the Labour Force Survey suggest a more acute contraction in hours worked (which will reflect that those people furloughed should not actually be working) than in employee numbers, with average hours worked falling by around a fifth between the last week of April 2020 and the same week a year earlier.[6]

If that gives a sense of scale, then what can we say about the distribution of these effects? Gardiner and Slaughter (2020), who analyse an online survey of 6,000 working-age adults from May 2020, find that 36 per cent of workers in the bottom quintile of pre-coronavirus earnings have experienced job loss, furloughing, or cuts in hours and earnings compared with 16 per cent in the top quintile. Both they and Adams-Prassl *et al*. (2020) (who draw on a separate online survey fielded in April 2020) show that these outcomes have been more common for those with atypical work arrangements (particularly temporary workers in the case of job loss; and non-salaried, zero-hours contract, and variable hours workers in the case of furloughing). These findings are broadly replicated in Benzeval *et al*. (2020), who find large and significant reductions in average working hours for women, likely to be reflecting at least in part the disproportionate additional childcare and home schooling hours working mothers have taken on in the face of school and nursery closures (Andrew *et al*., 2020). And both Benzeval *et al*. (2020) and Gustafsson (2020) suggest that employment impacts have been concentrated among the youngest and oldest workers. Of course, these findings hide heterogeneity in outcomes among the groups mentioned, reflecting variations between sectors and the ability to work from

[6] Claimant count data is in ONS (2020*a*). Hours worked each week is in ONS (2020*c*). Data from PAYE systems is reported in ONS (2020*b*).

home (Gustafsson and McCurdy, 2020; Joyce and Xu, 2020), as well as the fact that designated 'key workers' (meaning those in jobs which had to continue even when the lockdown was at its most severe) are more likely to be in the bottom half of earners than the top half (Farquharson *et al.*, 2020; Gustafsson and McCurdy, 2020). But the emerging conclusion is that the labour market shock has, so far, hit lower earners much harder than the better paid.

There is, though, a difference between having low earnings and being on a low income. Brewer and Gardiner (2020) show that the incidence of job loss or reductions in earnings is more common in the second and third quintiles of the working-age income distribution than in the bottom quintile, because around half of adults in the bottom quintile were not in work before the pandemic began. Similarly, Benzeval *et al*. (2020, Table 5) show that the pattern of declines in earnings—which are greater for low-income households than high-income households—are flatter when looking at all households, compared to when restricted just to households who had some earnings pre-pandemic. The conclusion that 'falls in market income affect only those engaged with the market' is uncontroversial, but it is important when considering the impact on low-income households.

### 3.2. The direct impact of the government's coronavirus measures

We consider here the three major measures in turn.

Broadly speaking, and relative to a counterfactual of job loss, the JRS will benefit those employees whose employers furloughed them. The fact that support via the JRS is based on a percentage of prior earnings up to a high cap will mean that higher earners who have been furloughed benefit most in absolute terms, but less as a fraction of previous earnings. Survey data suggest that being furloughed is more common among low earners than high earners (Figure 1 of Gardiner and Slaughter (2020)), but that, among all (working-age) adults, furloughing has been most common in the middle of the (pre-crisis) household income distribution (see Figure 3 of Brewer and Gardiner (2020)); this is because low earners are not found only in low-income households. Brewer and Tasseva (2020, Figure 1) estimate the impact of the JRS on household incomes, assuming that the counterfactual is that the worker would have no earnings at all, and find that the impact of the JRS is slightly greater, as a percentage of disposable income, in (pre-crisis) income decile groups 6–9 than it is in 2–5, but that its impact tails off in the top decile group because of the £2,500 per month cap, and is much smaller in the lowest-income decile group because fewer adults were in work pre-crisis (Bronka *et al.* (2020, Figure 3) have similar conclusions). This suggests that the distributional impact of the JRS, when considered across all individuals, is currently neutral or slightly regressive (as in, pro-rich).

At the time of writing, there were few data on who was claiming the SEISS (but Blundell and Machin (2020) report on the extent to which self-employed workers' businesses are affected by the pandemic and on what sort of self-employed workers said that they intended to claim it). Self-employment is most common at the very bottom and very top of the household income distribution, but the restrictions to SEISS access will be likely to affect both of these groups, with those on lower incomes less likely to have filed tax returns, and some of those on higher incomes missing out due to high profits or incorporation, so it is possible that the greatest impact of this scheme will be felt in the middle of the income distribution.

The increased generosity of means-tested benefits announced in response to COVID-19 will help those who were receiving UC or tax credits before the crisis, as well as the newly unemployed or those whose earnings have fallen who go on to claim UC. Brewer and Handscomb (2020) shows the 'overnight' distributional impact of these changes. As would be expected from an increase in means-tested support, the majority of the beneficiaries are in the bottom half of the income distribution and, on average, the changes provide a boost to family incomes of nearly 5 per cent in the bottom quartile (Brewer and Tasseva (2020, Figure 4) comes to a similar conclusion, although it expresses gains as a fraction of before-housing-costs income, meaning that the gains are shown to be a smaller fraction of income).

### 3.3. The overall impact on the income distribution

At the time of writing, there were only three studies that suggested how the crisis might be affecting the distribution of income. Brewer and Gardiner (2020) report results from a survey that asked working-age adults about changes in household income between February and May 2020. They show that the experience of a fall in income is distributed surprisingly evenly across the (pre-coronavirus) income distribution: 37 per cent of adults in the bottom 40 per cent of working-age family-level incomes reported income falls, as did 35 per cent of adults in the top 40 per cent. Indeed, experience of increases in income was actually most common among adults at the bottom of the pre-coronavirus income distribution; this could be due to the increases in benefit entitlement.

This study did not quantify the scale of the income changes. But two studies have used nowcasting techniques to simulate the distributional impact of the crisis by estimating the distribution of household incomes and comparing that to a counterfactual world where the crisis did not happen. These are: Brewer and Tasseva (2020), who use the data on job loss and falls in earnings analysed in Benzeval *et al*. (2020) to estimate how the crisis has affected household incomes in April 2020, and Bronka *et al*. (2020), who use data collected from an expert survey of 2,644 economists to estimate how the crisis has affected incomes in the full financial year 2020/21.

Both studies estimate, on average, that the crisis reduces household incomes, and the falls in income are greater in proportional terms for households who would have been at the top of the income distribution. However, the estimated impacts vary markedly, with Brewer and Tasseva (2020) forecasting a 9 per cent fall, and Bronka *et al*. (2020) a 1 per cent fall. The most likely explanation is that the former paper is an assessment of how COVID-19 is affecting incomes in April 2020 only, and the latter is making an assessment over the 2020/21 financial year. Poverty rates against a fixed poverty line are simulated to rise in Brewer and Tasseva (2020)'s analysis (from 17.4 to 19.8 per cent; Table A.4).

### 3.4. The impact on wider measures of household living standards

To come to a full understanding of the impact of the current crisis on living standards across the distribution, and poverty, it is helpful also to go beyond measures of living standards other than income.

Figure 3, from Brewer and Gardiner (2020), shows how adults report both income and spending to have changed between February and May 2020, and find a strong distributional gradient: 38 per cent of adults in the top (pre-coronavirus, working-age) income quintile have experienced no income falls but have had a reduction in spending compared to just 12 per cent of those in the bottom quintile. Consumption is often viewed as an alternative and more detailed lens on living standards, compared to income and income-based poverty measures (see Brewer *et al*. (2017) and Gardiner *et al*. (2017), for example), and in normal times we would usually interpret falls in spending as indicative of a decline in household resources. Here, it seems likely that the fall in spending reflects more an inability to spend money, given that much of the hospitality, non-food retail, and leisure sectors had been shut down from late March to May 2020. If so, then, for many families, especially the better off, falls in spending probably reflect 'enforced saving' (Corry (2020) shows that better-off families devoted more of their spending to the sectors that were shut down). This is matched by subjective data: Brewer and Gardiner (2020) report that adults in the (pre-coronavirus, working-age) top quintile were as likely to say that their personal financial situation has improved as worsened (23 per cent compared to 22 per cent), whereas in the bottom quintile the figures are 10 per cent vs 36 per cent. Adults in the (pre-coronavirus, working-age) bottom quintile were also more likely to say that they were now more concerned about finances than they were before the crisis than were adults in the top quintile. And these results are matched by analysis in Bangham and Leslie (2020), which show that low-income working-age families are more likely (than high-income families) to have reduced their saving rate, or increased their use of consumer debt, or borrowed from friends and family. Benzeval *et al*. (2020) also find the same patterns of mitigation behaviours among those whose earnings have fallen, with running down savings being most common in the bottom income quintile group, and borrowing more or receiving loans from family and friends being most common in the bottom two quintile groups. Overall, data so far strongly suggest that high-income families are more likely to have seen a strengthening of the household's financial position, and that the implications of this crisis are more serious for the living standards of lower-income (working-age) families than higher-income ones.

**Figure 3:** Change in household income and spending compared to before the coronavirus outbreak began, by 18–65-year-old family income quintile before coronavirus (exc. retired and students): UK, 6–11 May 2020

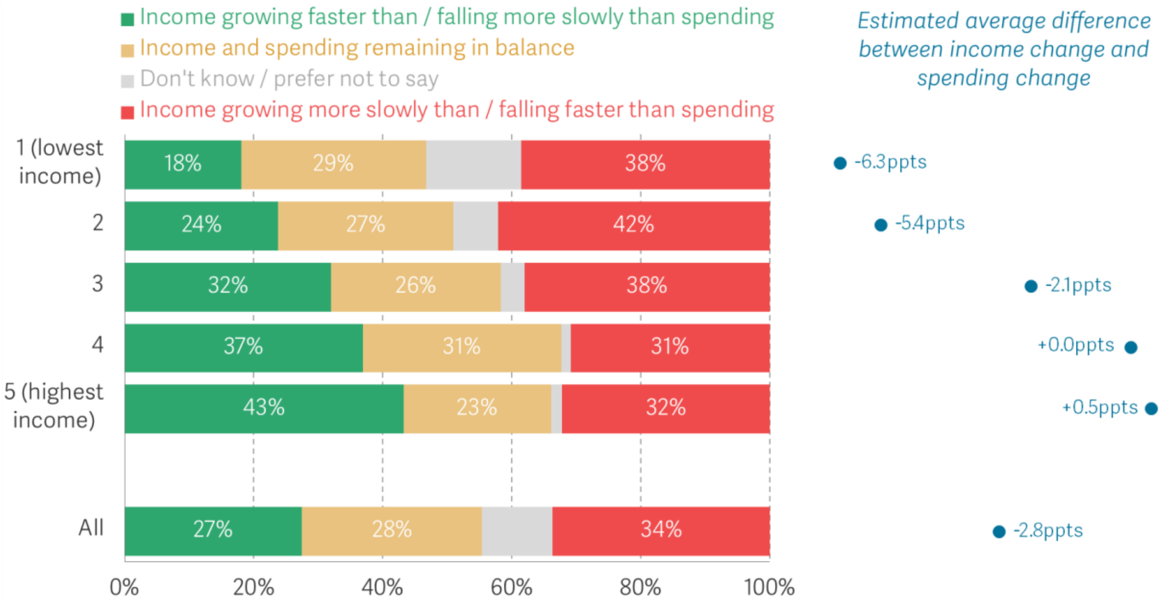

*Notes*: Base = all adults aged 18–65 with valid income data (apart from the 'all' category). Family income distribution based on equivalized, disposable benefit unit incomes among 18–65-year-old adults, excluding families containing retired adults or non-working adult students (see the annex to Brewer and Gardiner (2020) for details). Right-hand panel excludes those responding 'don't know' or 'prefer not to say', and is based on assigning those in income and spending change categories values as follows: +/–>25 per cent = 33 per cent; +/–10–25 per cent = 17.5 per cent; +/–<10 per cent = 5 per cent.

*Source*: Brewer and Gardiner (2020).

## 4. Summary and conclusions

This article has analysed the UK government's three main policies to protect directly household incomes during the coronavirus crisis. We have also considered the emerging evidence on the overall impact of the pandemic on the living standards of low-income households, given what is known at the time of writing about the distributional pattern of the labour market shock and the major policy responses.

So far, it seems that a bottom-heavy labour market hit has not translated into as bottom-heavy a hit to family incomes, partly due to the success of the (strengthened) social security safety net in cushioning the blow—at least to some extent, and for some—and partly because many at the bottom of the income distribution are unaffected by job loss. On the other hand, looking at wider measures of living standards reveals a more concerning picture, with those in (pre-crisis) lower-income families being far more likely to have taken on new debt, or borrowed from friends or family, or cut back on saving; this reflects not that the income falls have been greater than among higher-income households but that they have been less likely to have experienced a (probably enforced) reduction in spending. This, in addition to evidence on people's assessments of their own situation, suggests that the crisis is having more marked negative effects on the living standards—considered broadly—of lower-income working-age families than of higher-income families. Furthermore, this comes after several years where incomes have grown very slowly at the bottom of the income distribution, particularly measuring incomes after housing costs have been deducted (Bourquin *et al.*, 2020).

Most of the evidence to date comes from surveys of working-age adults. We surmise, though, that those above working age will be less affected, in financial terms, by the coronavirus crisis than those of working age. Lower-income people over the pension age in UK get the majority of their income from the government in social security benefits, rather than the labour market. And although asset prices have fallen and interest rates are very low (both of which tend to reduce the income that people can get from their investments), this crisis is not rooted in the financial markets, as was the recession after the global financial crisis.

These findings offer important lessons for policy-makers. As the government considers how policies should develop over the recovery phase of this crisis—including what (if anything) should replace the Coronavirus Job Retention Scheme after October 2020, the introduction of support to help unemployed people return to work, and any extension of the currently temporary £20 per week uplift to Universal Credit or other changes to welfare benefits—policy-makers should be particularly focused on providing support to those on lower incomes where the changes in overall financial circumstances have been the most concerning so far. Further down the track, any fiscal consolidation after this crisis that is needed to reduce the government's deficit should be designed with an eye to those whose overall financial situation has been least affected by the crisis. Appreciating the differential impacts of this crisis on different groups within society will allow policy-makers to support the recovery most effectively, and prevent the coronavirus crisis, as far as is possible, from becoming a long-term crisis in living standards.
